# Diffuse Gallium-67 Accumulation in the Left Atrial Wall Detected Using SPECT/CT Fusion Images

**DOI:** 10.1155/2016/6374584

**Published:** 2016-12-20

**Authors:** Kohei Kotani, Joji Kawabe, Shigeaki Higashiyama, Atsushi Yoshida, Susumu Shiomi

**Affiliations:** Department of Nuclear Medicine, Graduate School of Medicine, Osaka City University, 1-4-3 Asahi-machi, Abeno-ku, Osaka 545-8585, Japan

## Abstract

Gallium-67 scintigraphy is useful for detecting active inflammation. We show a 66-year-old female patient with atrial fibrillation and diffuse thickening of the left atrial wall due to acute myocarditis, who presented diffuse abnormal accumulation of gallium-67 in the left atrium on single photon emission computed tomography/computed tomography (SPECT/CT) fusion images. In the second gallium-67 scan 2 months after the first scintigraphy, the abnormal accumulation in the heart was no longer visible. Gallium-67 SPECT/CT images helped understanding the disease condition that temporary inflammation in the left atrium caused atrial fibrillation.

## 1. Introduction

Gallium-67 citrate injected intravenously binds to transferrin, and it is incorporated into the transferrin receptor of inflammatory cells or malignant tumor cells. Because of the above-mentioned properties, gallium-67 scintigraphy is used to detect a focus of active inflammation or malignant lesion. In Japan, F18-fluorodeoxyglucose positron emission tomography (FDG-PET) examination is now not reimbursed by Japanese health insurance system for the diagnosis of active inflammation except for cardiac sarcoidosis, but, globally, FDG-PET is more frequently used for the diagnosis of active inflammation and malignant disease because of its superior spatial resolution. Regarding heart disease, however, prolonged fasting should be required before FDG-PET to reduce physiological FDG accumulation in the heart. Gallium-67 scintigraphy is useful for examination of heart disease including cardiac sarcoidosis and acute myocarditis because gallium-67 does not accumulate physiologically in the heart [1, 2]. Herein, we report the case of a patient with diffuse thickening of the left atrial wall, in which gallium-67 scintigraphy helped understanding the disease condition.

## 2. Case Report

**Figure 1:**
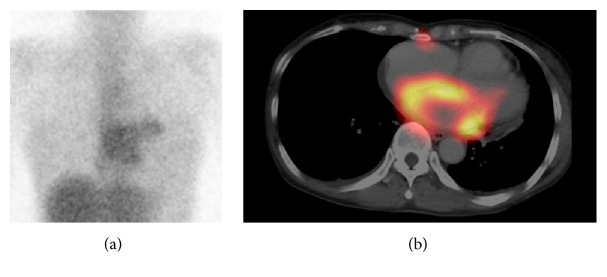
Gallium-67 imaging was performed 72 hours after intravenous injection with 74 MBq of gallium-67 citrate. (a) Frontal planar image of gallium-67 scan showed abnormal accumulation of radioisotope in the heart. (b) Single photon emission computed tomography/computed tomography fusion image of gallium-67 scan showed diffuse abnormal accumulation of radioisotope in the thickened left atrial wall.

A 66-year-old woman visited our hospital with a chief complaint of palpitations. Since electrocardiogram showed paroxysmal atrial fibrillation and echocardiography showed a thrombus in the left atrium, she was admitted to receive treatment. Echocardiography also showed pericardial effusion and circumferential thickening of the left atrial wall. The thrombus in the left atrium revealed high echoic mass, while circumferential thickening of the left atrial wall revealed low echoic lesion in echocardiography. Thus, these two parts were completely different components. To examine active inflammation or malignancy for the thickened left atrial wall, gallium-67 scintigraphy was performed. The frontal planar image showed abnormal accumulation of radioisotope (RI) in the chest (Figure 1(a)). The single photon emission computed tomography/computed tomography (SPECT/CT) fusion images showed corresponding diffuse abnormal accumulation of RI in the thickened left atrial wall (Figure 1(b)), possibly suggesting active inflammation in the left atrial wall including acute myocarditis, sarcoidosis, or amyloidosis or malignant disease such as malignant lymphoma. However, clinical symptoms and subsequent general examination showed no findings suggestive of sarcoidosis, amyloidosis, or malignant lymphoma. As the patient had no increased white blood cell count and C-reactive protein level and had no symptoms other than palpitations, she was followed up without receiving specific treatment for the atrial lesion.

**Figure 2:**
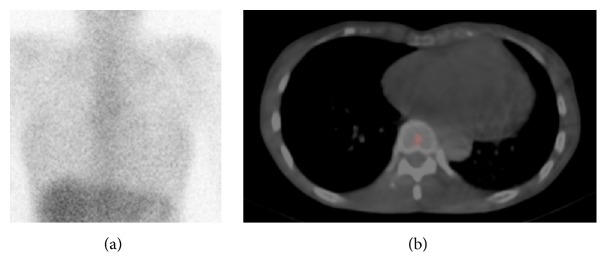
The second gallium-67 scintigraphy performed 2 months after the first scintigraphy. Gallium-67 imaging was performed 72 hours after intravenous injection with 74 MBq of gallium-67 citrate. (a) Frontal planar image of gallium-67 scan showed no abnormal accumulation of radioisotope in the heart. (b) Single photon emission computed tomography/computed tomography image of gallium-67 scan also showed no abnormal accumulation of radioisotope in the left atrial wall.

Anticoagulant therapy for the thrombus in the left atrium resulted in its dissolution, and administration of a *β*-blocker for atrial fibrillation achieved favorable heart rate control. In the second gallium-67 scan 2 months after the first scintigraphy, the abnormal accumulation in the heart was no longer visible on the planar and SPECT/CT images (Figure 2). Echocardiography showed no thickening of the left atrial wall. Regarding the cause of cardiac inflammation, acute myocarditis was considered because the serum antibody titer of cytomegalovirus was significantly elevated in a few weeks though endomyocardial biopsy was not done because the patient did not agree with it. She is now continuing follow-up check of atrial fibrillation in outpatient department.

## 3. Discussion

Gallium-67 scintigraphy is useful for detecting active inflammation or malignant lesion. Regarding cardiac disease, it plays an important role in examining inflammatory disease including acute myocarditis, cardiac sarcoidosis, and cardiac amyloidosis [1–3].

Several studies report the gallium-67 scan to be useful in the diagnosis of acute myocarditis [1, 4]. The gold standard of diagnosis for acute myocarditis remains endomyocardial biopsy though the diagnostic accuracy of this procedure is limited [5, 6]. In clinical settings, the diagnosis of acute myocarditis is often judged by a clinical examination because endomyocardial biopsy has a risk of bleeding and cardiac tamponade. In our case, first and second gallium-67 SPECT/CT images clearly demonstrated that left atrial inflammation improved as time went by. Therefore, endomyocardial biopsy was not performed though biopsy was considered for definite diagnosis.

Some reports examined accumulation of RI in cardiac sarcoidosis lesions using gallium-67 SPECT/CT and FDG-PET/CT [7, 8], but these lesions are seen in the left ventricular wall in general. Till date, there is no report confirming localized gallium-67 accumulation only in the left atrium using SPECT/CT imaging. In the case of acute myocarditis, diffuse inflammation in the myocardium mainly on the left ventricle is seen. However, a case in which diffuse FDG uptake was seen in the left atrium due to myocarditis confined to the left atrium had been reported [9]. In addition, a case in which diffuse FDG uptake was seen in the left atrium due to atrial fibrillation had been reported [10]. Referring to previous reports, FDG may accumulate in the myocardium because of hyperenergia of glucose metabolism due to myocardial load besides inflammatory change. On the other hand, gallium-67 accumulates in the myocardium because of not myocardial load but inflammatory change. In our case, we speculated that gallium-67 SPECT/CT revealed diffuse inflammation in the left atrium, which was useful for understanding that temporary inflammation due to confined myocarditis in the left atrium caused paroxysmal atrial fibrillation. Recently, associations between cardiac inflammation and atrial fibrillation have been reported [11], and inflammatory cells such as macrophages are recruited across atrial endocardium in atrial fibrillation [12]. We think gallium-67 accumulation in the left atrium is due to acute myocarditis because the serum antibody titer of cytomegalovirus was elevated in a few weeks, but we cannot deny the possibility that it is due to an idiopathic infiltration of inflammatory cells, which is considered to be one of the causes of atrial fibrillation. Further clinical case series are needed for verifying this possibility.

## 4. Conclusion

This patient presented with atrial fibrillation on the electrocardiogram, left atrial thrombosis, and diffuse thickening of the left atrial wall on the echocardiography. SPECT/CT fusion images of gallium-67 scan showed corresponding diffuse abnormal accumulation of RI in the left atrial wall. These findings were useful for the localization of inflammation due to acute myocarditis. When abnormal accumulation of gallium-67 is observed in the heart on planar images, an additional SPECT/CT scan may be useful for evaluation of accurate localization.
